# Prevalence, Genetic Diversity and Factors Associated with Distribution of *Listeria monocytogenes* and Other *Listeria* spp. in Cattle Farms in Latvia

**DOI:** 10.3390/pathogens10070851

**Published:** 2021-07-06

**Authors:** Margarita Terentjeva, Žanete Šteingolde, Irēna Meistere, Didzis Elferts, Jeļena Avsejenko, Madara Streikiša, Silva Gradovska, Laura Alksne, Juris Ķibilds, Aivars Bērziņš

**Affiliations:** 1 Institute of Food and Environmental Hygiene, Faculty of Veterinary Medicine, Latvia University of Life Sciences and Technologies, LV-3004 Jelgava, Latvia; zanete.steingolde@bior.lv (Ž.Š.); aivars.berzins@bior.lv (A.B.); 2 Institute of Food Safety, Animal Health and Environment “BIOR”, LV-1076 Rīga, Latvia; irena.meistere@bior.lv (I.M.); jelena.avsejenko@bior.lv (J.A.); madara.streikisa@bior.lv (M.S.); silva.gradovska@bior.lv (S.G.); laura.alksne@bior.lv (L.A.); juris.kibilds@bior.lv (J.Ķ.); 3 Faculty of Biology, University of Latvia, LV-1004 Rīga, Latvia; didzis.elferts@lu.lv

**Keywords:** serogroups, clonal complexes, feed, soil, water, feces, epidemiology, WGS, Latvia

## Abstract

*Listeria* spp. is a diverse genus of Gram-positive bacteria commonly present in the environment while *L. monocytogenes* and *L. ivanovii* are well known human and ruminant pathogens. The aim of the present study was to reveal the prevalence and genetic diversity of *L. monocytogenes* and other *Listeria* spp. and to identify the factors related to the abundance of pathogen at cattle farms. A total of 521 animal and environmental samples from 27 meat and dairy cattle farms were investigated and the genetic diversity of *L. monocytogenes* isolates was studied with WGS. The prevalence of *Listeria* was 58.9%, while of *L. monocytogenes* it was −11%. The highest prevalence of *L. monocytogenes* was found in the environment—soil samples near to manure storage (93%), mixed feed from the feeding trough and hay (29%), water samples from farms drinking trough (28%) and cattle feces (28%). Clonal complexes (CC) of CC37 (30%), CC11 (20%) and CC18 (17%) (all IIa serogroup) were predominant *L. monocytogenes* clones. CC18, CC37 and CC8 were isolated from case farms and CC37, CC11 and CC18 from farms without listeriosis history. Only one hypervirulent CC4 (1%) was isolated from the case farm. Sequence types (STs) were not associated with the isolation source, except for ST7, which was significantly associated with soil (*p* < 0.05). The contamination of soil, feeding tables and troughs with *L. monocytogenes* was associated with an increased prevalence of *L. monocytogenes* at farms. Our study indicates the importance of hygienic practice in the prevention of the dissemination of *L. monocytogenes* in the cattle farm environment.

## 1. Introduction

Genus *Listeria* consists of 21 species of which *Listeria monocytogenes* and *L. ivanovii* were found to be pathogenic [1]. While *L. ivanovii* is associated with animal infection, *L. monocytogenes* is responsible for listeriosis in humans and animals [2]. In humans, listeriosis is characterized with gastroenteritis or severe manifestations including central nervous system disorders, miscarriage and even death may occur in immunocompromised individuals [3]. *L. monocytogenes* is a foodborne pathogen and products that do not undergo sufficient thermal treatment to eliminate the pathogen or that are consumed without any processing are considered to be high-risk foods. Outbreaks of listeriosis have been linked to the contamination of unpasteurized milk and milk products, soft cheeses, fish and seafood, ready-to-eat meat products with the growing importance of plant-based novel food vehicles [4,5,6]. Foods with *L. monocytogenes* may become contaminated before or during processing due to the occurrence and/or persistence of the pathogen in the animal farms and the food-processing environment [7]. 

Since the same *L. monocytogenes* genotypes were found in animals, farms and food producing environments, it has been assumed that the dairy cattle may serve as a source of milk contamination with public health implications [8]. *L. monocytogenes* primarily affects ruminants and may lead to significant economic losses. A typical manifestation of listeriosis in animals includes encephalitis and septicemia, which may cause fetal infection and abortion [9,10]. Listeriosis outbreaks in ruminants were attributed to the consumption of silage, which may become contaminated from external environments including plants and soil, water, manure and wildlife due to the widespread prevalence of *Listeria* spp. and *L. monocytogenes* in the environment [11,12]. However, the transmission of *L. monocytogenes* in farms and their importance for animal and public health is still not well understood [8]. 

*Listeria* spp. and *L. monocytogenes* were isolated from the farm and surrounding environment—water, soil and feed—indicating a variety of different sources of contamination [8,13]. Ruminants are frequently identified as asymptomatic carriers of *L. monocytogenes* that can directly excrete the pathogen [14,15,16]. This may contribute to the spread of infection between animals and the dissemination of the pathogen in the farm environment. Studies on the diversity of *L. monocytogenes* in animals and the environment may help to better understand the epidemiology of listeriosis in the ruminants. 

The characterization of clinical and environmental isolates of *L. monocytogenes* is important since the circulation of the same type within farms or geographic regions was reported. In dairy cattle, *L. monocytogenes* genotypes associated with human outbreaks were found; therefore, the characterization of *Listeria* isolates has significant public health importance [17,18]. Novel typing methods, such as whole-genome sequencing (WGS), including core genome multilocus sequence typing (cgMLST), can successfully be applied to analyze the origins and contamination sources of *L. monocytogenes*. Until now, cgMLST showed s higher discriminatory accuracy compared to the pulsed-field gel electrophoresis (PFGE) method and multilocus sequence typing (MLST) that may provide new data on the epidemiology of *L. monocytogenes* from an animal and public health perspective [19]. 

WGS and cgMLST-based typing allows the genetic diversity of *L. monocytogenes* isolates to be characterized by sequence type (ST) and clonal complex (CC) that shows considerable differences in ecology, virulence and the clinical potential of the pathogenic isolates [19,20]. Hypervirulent clones have been reported to be more effective in the colonization of the intestinal tract accompanied with the wider dissemination of the pathogen in the organisms. CC1, CC2, CC4 and CC6 were reported to be associated with a clinical origin [19,20]. Hypervirulent CC1, CC4-CC127 were linked to rhombencephalitis and abortus in ruminants, while a high prevalence of CC2, CC4 and CC11 was found in subclinical cattle mastitis that potentially may serve as milk contaminants [21,22]. CC9 and CC121 are food-associated hypovirulent MLST clones which were implicated in listeriosis in immunocompromised individuals [19,23]. The reported prevalence of hypervirulent clones in animals, farm environments and foods highlights the importance of understanding the ecology and transmission of *L. monocytogenes* [16,21,24]. Studies on the prevalence and genetic diversity of *L. monocytogenes* in the cattle farm environment may provide a new insight on the epidemiology of *L. monocytogenes*. The aim of the present study was to study the prevalence of *Listeria* spp. and *L. monocytogenes* in the environment of cattle farms, characterize the genetic diversity of *L. monocytogenes* and to identify the risk factors related to clinical listeriosis in ruminant farms.

## 2. Results

### 2.1. Prevalence of Listeria spp. in the Farm Environment

Altogether six *Listeria* species were identified in environmental samples from farms with a prevalence of 58.9% (307/521). *L. monocytogenes*, *L. innocua* and *L. seeligeri* were isolated from all types of samples. *L. fleishmanii* was sporadically found in soil and feed, *L. welshimeri* in feed and water but *L. ivanovii* was found in water and animal feces (Table 1). The highest prevalence of *L. monocytogenes* was found in feces—25.2%, while the lowest was in feed—14.9%. The prevalence of *L. monocytogenes* at individual farms varied from 0 to 80%. The prevalence of *L. monocytogenes* of 33% in animals at case farms (11/33) was higher than the prevalence of 22% identified at control farms (17/78). The prevalence of *L. innocua* was significantly higher than the prevalence of other Listeria species (*p* < 0.001).

The highest prevalence of *L. monocytogenes*—29%—was identified in the mixed feed from the feeding trough and hay, while the lowest prevalence of 4% was found in silage. The highest prevalence of *L. innocua* of 65% was found in the mixed feed from the feeding trough, while the lowest prevalence of *L. innocua* of 14% was revealed in silage (Table 2). The highest prevalence of *L. seeligeri* of 19% was observed in hay, but the lowest prevalence of 4% was identified in grains and flours. *L. monocytogenes* counts were from 1.48 log cfu/g in the total mixed ratio (TMR) to 5.15 log cfu/g in the feed from the feeding troughs. *L. innocua* counts varied from 2.04 log cfu/g in grass to 5.77 log cfu/g in silage. *L. seeligeri* counts ranged from 1.83 log cfu/g in hay to 6.06 log cfu/g in silage. The significant differences between the mean counts of *L. monocytogenes*, *L. innocua* and *L. seeligeri* in different types of feeds were not identified (*p* > 0.05). 

The highest prevalence of *L. monocytogenes* of 93% was identified in soil near to the manure storage, but the lowest of 33% near to the pond (*p* < 0.001). The highest prevalence of *L. innocua* (67%) was found near to the manure storage, while the lowest prevalence of *L. innocua* of 17% was at the territory of farms. The highest prevalence of *L. seeligeri* of 20% was found near to manure storage, but the lowest prevalence of 5% was found at the pasture (Table 3). In feed, the highest prevalence of *L. monocytogenes* (24%), *L. innocua* (51%) and *L. seeligeri* (8%) was identified in feed from feed tables and troughs, while the lowest prevalence was found at the pastures—0%, 33% and 0%, respectively. In water, the highest prevalence of *L. monocytogenes* of 48% was identified in water bodies at pasture, but the lowest of 10% was in drinking troughs. The highest prevalence of *L. innocua* of 60% was found in drinking bowls, while the lowest of 12% was in other sources (ditch). The highest prevalence of *L. seeligeri* of 28% was found in water bodies, but the lowest of 8% was in drinking bowls. Other *Listeria* species associated with water from the farm environment were *L. welshimeri* and *L. ivanovii* (1.5%) isolated from the river and the farm water. 

### 2.2. Molecular Serotyping, Clonal Complexes (CCs) and Genetic Diversity of Listeria Species Isolated from the Farm Environment 

At least one *L. monocytogenes* representative for each farm and each source type was selected for sequencing and, after quality control, 67 sequences were included in further characterization. The majority of the sequenced *L. monocytogenes* isolates were of the IIa serogroup (64 out of 67), two isolates were IVb and one isolate IIc. The serogroup IIa was detected in various sources—soil, feed, water and animal feces—while IVb was in water and feces, but IIc was only in feces (Table 3). 

Altogether 15 STs/15 CCs were detected in *L. monocytogenes* isolates with multilocus sequence typing. In total, the most abundant STs (CCs) were ST37 (CC37) (30%, 20/67), ST451 (CC11) (20%, 13/67) and ST18 (CC18) (17%, 11/67) (Table 4). 

Between the analyzed *L. monocytogenes* isolates, the predominant STs (CCs) at case farms were ST18 (CC18) (24%, 11/33), ST37 (CC37) (21%, 7/33) and ST (CC8) (18%, 6/33), but at control farms they were ST37 (CC37) (38%, 13/34), ST451 (CC11) (24%, 8/34) and ST18 (CC18) (9%, 3/34) (Table 5). There were not significant differences in the prevalence of the most abundant STs/CCs between the case and control farms (*p* > 0.05). 

**Figure 1:**
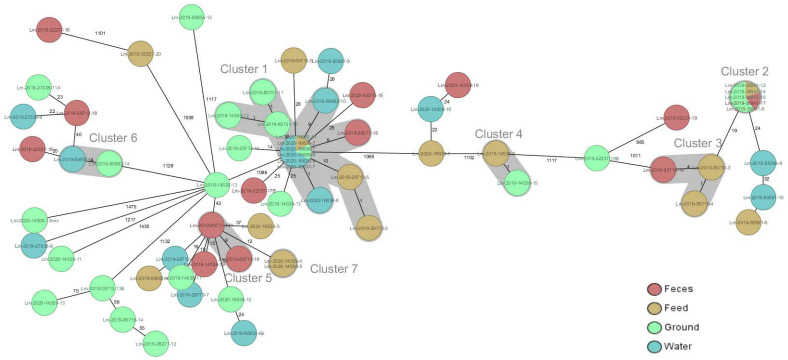
Ridom SeqSphere + Minimum spanning tree of 67 *L. monocytogenes* isolates from cattle farms based on *L. monocytogenes* 1701 cgMLST loci pairwise ignoring missing values. For each isolated farm number and ST that is indicated, the source of isolate is represented by color code and the number on lines represent the distance between isolates in an absolute number of loci. Cluster alert distance—10 loci.

Between the isolates limited to one farm from one up to six different STs (CCs) were observed. Most of the STs (CCs) were not associated with certain sources, except for ST7 (CC7) that was significantly associated with soil and was observed only between soil isolates from four different farms (*p* > 0.05) (Table 4, Figure 1). Comparing isolates at a cgMLST level (Figure 1), a total of seven clusters were observed with six of them (clusters 2, 3, 4, 5, 6, 7) were limited to a single farm including two to five isolates from various sources. The distances between isolates within clusters were 0–9 alleles. Cluster 1 of ST37 (CC37) included 14 isolates from seven farms. Within Cluster 1, the distance between the isolates limited to one farm was smaller (0–1 allele) than between different farms (0–10 alleles) (Figure 1). 

### 2.3. Factors Associated with L. monocytogenes within-Farm Prevalence

A lack of cleaning and disinfection of the feeding tables was associated with an increased prevalence of *L. monocytogenes* in soil samples of case farms (odds ratio: 3.89, 95% credibility interval: 1.11–37.31) and control farms (odds ratio: 2.56, 95% credibility interval: 1.09–9.98). The contamination of water samples with *L. monocytogenes* was associated with the type of production—beef farms had an increased prevalence of *L. monocytogenes* (odds ratio: 3.29, 95% credibility interval: 1.12–9.87). In this study, there were no significant differences in the association for all the other models.

## 3. Discussion

*Listeria* spp. was isolated from the farm environment where the highest prevalence of *L. monocytogenes* was found in cattle feces (25%). Overall, the prevalence of *L. monocytogenes* was comparable with previous studies confirming that the cattle may serve as a significant reservoir of *L. monocytogenes* [8,25]. *L. monocytogenes* was identified in farms without listeriosis records, which indicates that animals may shed the pathogen asymptomatically and excrete *L. monocytogenes* in the farm environment, as was proposed previously [14,16,18]. The higher prevalence of *L. monocytogenes* in cattle from case farms than in control farms was in agreement with previous reports [14,26]. 

Hay and animal feed in feed bunks were found to be the most contaminated with *L. monocytogenes* (29%), which was higher than that reported by Fox et al. [27]. In contrast, the identified prevalence of *L. monocytogenes* in silage (4%), which is supposed to be the main source of the pathogen for cattle, was lower than the 6.2–30% reported previously [8,13,28,29]. Non-*L. monocytogenes* species were more abundant in baled silage in comparison with *L. monocytogenes*, which is in line with previous reports [13,30]. Although in case farms baled silage was used, *L. monocytogenes* was rarely isolated. Silage that suffered aerobic spoilage may harbor *Listeria* species due to the favorable conditions for the survival and growth of *Listeria* spp., including pathogenic ones [30]. The low density of silage, the high pH and the presence of oxygen due to bag damage or an insufficient amount of the plastic were linked to the growth of *L. monocytogenes* in baled silage [12,31]. 

*L. monocytogenes* was also isolated from soil, water and feed samples, which indicates that animals can be exposed to different sources of contamination. The farm environment was found to be contaminated with *L. monocytogenes* due to close contact with the animals; however, the significant differences between the inside and outside environment of the farms were not identified in the water and soil samples (*p* > 0.05) with the exception of contaminated feed in feed troughs (*p* < 0.05). Within the individual sampling site, the highest prevalence of *L. monocytogenes* was found in soil near to the farm (38%), the feed trough and the drinking trough (28%). Our results are in accordance with published studies on the ecology of *L. monocytogenes* in the farm environment [8,13]. The high prevalence and colonization of feed bunks and water troughs with *L. monocytogenes* could contribute to the exposure of cattle to the pathogen [8,13]. The fecal shedding and following spread of *L. monocytogenes* to the surrounding environment could be the main source of infection for animals at farms. 

Several *L. monocytogenes* clones were observed in all the studied farms. The multitude of different contamination sources with *L. monocytogenes* in the farm environment may lead to the higher diversity of STs (CCs) identified in the cattle fecal isolates due to the exposure to the pathogen from in-farm contamination sites [32]. Widespread distribution of the same CCs in the internal and external environment of the cattle farms supports the hypothesis that cattle act as an important reservoir of *L. monocytogenes*. Thus, some specific measures, including animal and environmental hygiene, have to be considered to minimize unnecessary *Listeria* contamination and following growth in the farm environment.

In this study, the majority of CCs from case farms belonged to CC 8, CC11, CC18 and CC37 (all serogroup IIa). CC8, CC9 and CC11 were associated with food and persistence in food-processing environments, and were involved in listeriosis outbreaks [33,34]. CC8 and CC9 were previously identified in meat samples from broad geographical regions [35,36]. CC8 (IIa serogroup) was associated with a higher fatality rate and invasive human listeriosis cases in Poland and was described for the first time in association with the Canadian listeriosis outbreak in 1990–2010s [37,38]. 

The widespread distribution of *L. monocytogenes* CC18 and CC37 in control and case farms without significant differences in their prevalence (*p* > 0.05) may be associated with their environmental origin. In previous studies, ST37 (CC37) was associated with ruminants, ruminant farms and wildlife environments [21,39]. CC37 was prevalent among clinical isolates from ruminant farms [40]. Significantly, a higher prevalence of CC18 and CC37 clones was associated with milk products [33,41]. An abundance of CC37 and CC18 clones may indicate their adaptation and persistence in the cattle farm environment, [33,41]. CC7’s association with foodborne outbreaks, prevalence in foods and animals was established in previous studies [34,40]. 

CC4 was the only hypervirulent clone that has been isolated in the present study from one case farm. Within lineage I, isolates from CC1 have been reported as a significant cause of listeriosis and rhombencephalitis in ruminants in central Europe that might be related to the hypervirulence of CC1 [21]. The prevalence of hypervirulent clones CC4 and CC6 was reported in clinical isolates from ruminant farms; that is similar to the CC reported in clinical isolates in humans [40,41]. CC6 was associated with abortion in ruminants [40]. The present study reveals the genetic diversity of *L. monocytogenes* isolates associated with case farms without the predominance of hypervirulent clones in the cattle and farm environments. The high prevalence of environment adapted clones, which were associated with milk and milk products, highlights the importance of the in-farm occurrence of *L. monocytogenes* for the possible transmission of STs (CCs) in the cattle farms and dairy producing chain. Improper hygienic practices, access to pasture with contaminated soil and shedding of *L. monocytogenes* were factors associated with the prevalence of *L. monocytogenes*. Hygienic practice and the disinfection of the feeding table was a significant factor associated with the increased prevalence of *L. monocytogenes* at control and case farms. In a study by Castro et al. [8], the surfaces of the feeding tables were among the most frequently contaminated with persistent *L. monocytogenes* that may facilitate the oral intake of the pathogen with food and water. Infrequent cleaning of the feeding bunk was significant for the increased prevalence of *L. monocytogenes* in milk tanks [42]. 

The association between the distribution of *L. monocytogenes* in soil and insufficient disinfection alongside with the widespread distribution of environmental CC indicates that the farm and outside environment may significantly influence the presence of *L. monocytogenes* at the farm. Significantly, the higher prevalence of *L. monocytogenes* at beef cattle farms could be attributed to free range production and contamination of the surrounding environment, including the farm and pastures. 

## 4. Materials and Methods

### 4.1. Sampling

Altogether 521 samples were collected from 27 cattle farms from March 2019 till August 2020 in Latvia. Farms were defined as case farms (*n* = 9) where ruminant listeriosis was reported within the last three years (2016–2019) and control farms (*n* = 18) without listeriosis history in the last three years. Overall, 15 to 21 samples were collected from each farm, including on-site samples from the farm’s inside and outside environment, forage and animals. Sampling sites of animal feed (*n* = 141) included silage and other forage at the storage site and in the feed trough. Water samples (*n* = 136) were obtained from drinking bowls, troughs and taps at the farm or/and from drinking troughs or water bodies at the pasture. Soil samples (*n* = 133) were collected from pastures, farm territory, bedding and manure. Samples from animals (*n* = 111) were collected from cows at farms. Samples were aseptically collected in sample transportation bags with an amount up to 200 g for each individual feed, water and soil sample. Animal samples were collected from rectum with sterile gloves.

### 4.2. Microbiological Testing and Confirmation of Listeria spp.

The isolation of *Listeria* spp. from samples was performed according to ISO-11290-1 (2017). For the isolation procedure, an amount of 25 g/mL of sample was enriched in ½ Frazer broth (Biolife, Monza, Italy) and incubated at 30 °C for 24 h. Then, 0.1 mL of enriched suspension was transferred into 10 mL of Frazer broth (all microbiological media—Biolife, Monza, Italy) and incubated at 37 °C for 24 h. A 10-microliter loop of sample suspension in Frazer broth was plated out onto two selective *Listeria* agars according to Ottaaviani and Agosti (ALOA) and OXFORD formulation (OXFORD agar), and incubated at 37 °C. The presence of characteristic colonies of *Listeria* spp. was checked after 24–48 h of incubation and typical colonies were small and round (0.5–1 mm in diameter) colonies in blue, green color or blue-green color, with/without opaque halo on ALOA medium and black, brown or olive color colonies on OXFORD medium. Presumptive colonies were subcultured onto sheep blood agar overnight and confirmed and identified with MALDI-TOF Biotyper (Bruker, Bremen, Germany).

### 4.3. Whole Genome Sequencing 

At least one isolate of each source type per farm was selected for WGS analysis. DNA from fresh culture was extracted with QIAamp DNA Mini Kit (Qiagen, Hilden, Germany), according to the manufacturer’s protocol and used for WGS. Nextera XT Library construction kit (Illumina) and Illumina MiSeq with 300 bp paired-end reads were used for the preparation of libraries and sequencing, respectively. Sequencing adapters and low-quality bases were trimmed from raw reads using Trimmomatic v0.38 [43]. *De novo* assembly of the trimmed reads was performed with SPAdes v3.14.0 [44]. For *L. monocytogenes* isolate characterization, in general, serotyping in silico and ST/CC with a seven gene multi-locus scheme developed by Ragon et al. [45] were used. For detailed genome characterization, a cgMLST typing scheme based on 1701 gene-by-gene comparison was used [46]. For serotyping, ST and cgMLST determination allele calling were performed by SeqSphere+ (Ridom, Münster, Germany) [47]. The newly identified cgMLST alleles were submitted to the nomenclature server (www.cgmlst.org) maintained by Ridom. After quality control (N50 > 10,000, genome size, average coverage > 30), a total of 67 *L. monocytogenes* sequence isolates were selected for further data analysis. 

### 4.4. Questionnaire

Case and control farms were included in the study after their consent. During the visit of farms, a set of samples were collected, and the questionnaire was filled in by interviewing the farm owner, farm manager or farm veterinarian (included in Supplementary Data). The questions covered by the questionnaire included type of production and farm characteristics (size, type, number of animals, origin if animals), management (access to pasture, bedding material, drinking and feeding regimes, manure) with an assessment of pasture and in-farm holding condition, origin of feed and feeding regimen and biosecurity (access of farm and surrounding environment, cleaning and disinfection procedures, control of rodents, pests and wildlife, other production and companion animal at holding). Additionally, the information about Listeria-infected animals, including clinical symptoms, was collected at case farms (Table S1).

### 4.5. Data Analysis

Differences in the prevalence of *Listeria* species, *L. monocytogenes* and CCs in feed, water, soil and animal samples were calculated with Fisher’s exact test (*p* < 0.05). Bayesian binary logistic generalized the linear mixed effects models, as implemented in software R 4.0.4. [48], and library brms [49] were used to test the associations between contamination of the environment (soil, feed, water and feces) (response variable), the farm status (case or control cattle farm) (independent variable) and farm characteristics/management (independent variable). There were multiple models developed where each contained contamination of the tested environment as a dependent variable, and status and the interaction between the farms’ characteristic variables as independent variables. As there were multiple observations per farm and the farm ID was used as a random factor in the model, for the Bayesian models the number of iterations was set to 2000 for each of the four chains. The Rhat values (all values were close to 1.00) were used to assess the convergence of the model. If there was a significant interaction effect in the model, Tukey’s adjusted pairwise comparison of estimated marginal means, as implemented in R library emmeans, [50] was used to compare groups. 

## 5. Conclusions

Our study confirms that despite the widespread occurrence of *Listeria* spp. and *L. monocytogenes* in the farm environment, the highest prevalence was associated with animals. The contamination rates and genetic characterization of *L. monocytogenes* indicates that animals may be the most important source of *L. monocytogenes*, causing the circulation of *L. monocytogenes* in the farm and outside environments with further exposure of animals to the pathogen. Improper hygienic practices were strongly associated with the case farms indicating the importance of hygiene measures for the prevention of the on-farm spread of *L. monocytogenes*. 

## Figures and Tables

**Table 1:** Prevalence of *Listeria* spp. in the cattle farm environment.

*Listeria* Species	Sample							
	Soil (*n* = 133)		Feed (*n* = 141)		Water (*n* = 136)		Feces (*n* = 111)	
	No. of Positive Samples (%)	95% CI	No. of Positive Samples (%)	95% CI	No. of Positive Samples (%)	95% CI	No. of Positive Samples (%)	95% CI
*L. monocytogenes*	25 (18.8)	11.9–25.6	21 (14.9)	9.5–21.9	26 (19.1)	12.9–26.7	28 (25.2)	17.5–34.4
*L. innocua* ^a^	72 (54.1)	45.3–62.8	51 (36.2)	28.3–44.7	53 (38.9)	30.7–47.7	37 (33.2)	24.7–42.9
*L. seeligeri*	14 (10.5)	5.8–17.0	11 (7.8)	3.9–13.5	11 (8.1)	4.1–14.0	17 (15.3)	9.2–23.4
*L. fleishmanii*	1 (0.8)	0.0–4.1	1 (0.8)	0.0–3.9	0 (0)	0	0 (0)	0
*L. welshimeri*	0 (0)	0.0–2.7	1 (0.8)	0.0–3.9	2 (1.5)	1.8–5.2	0 (0)	0
*L. ivanovii*	0 (0)	0.0–2.7	0 (0)	0	2 (1.5)	1.8–5.2	3 (2.7)	0.6–7.7
Total	93 (69.9)	61.3–77.6	71 (50.4)	41.8–58.9	78 (57.4)	48.6–65.8	65 (58.6)	48.8–67.8

^a^ Prevalence of *L. innocua* was significantly higher than the prevalence of other *Listeria* species (*p* < 0.001).

**Table 2:** Prevalence of *Listeria* spp. in animal feed at cattle farms in Latvia.

			*L. monocytogenes*					*L. innocua*		*L. seeligeri*	
Feed	No. of Samples	No. of *Listeria* Positive Samples (%)	No. of Positive Samples (%)	Serogroups	ST ^a^	CC ^b^	Mean log cfu/g	No. Positive Samples (%)	Cfu/g	No. of Positive Samples (%)	Cfu/g
TMR ^c^	5	3 (60)	1 (20)	ND	-	-	1.48	2 (40)	4.48	0 (0)	-
By-products ^d^	4	2 (50)	0 (0)	-	-	-	-	2 (50)	2.22	0 (0)	-
Pastures	3	1 (33)	0 (0)	-	-	-	-	1 (33)	2.04	0 (0)	-
Feed in feed trough	34	28 (82)	10 (29)	IIa, IVb	18, 20, 37, 194, 451		5.15	22 (65)	5.22	2 (6)	5.00
Hay	21	12 (57)	6 (29)	IIa	18, 37	18, 37	4.37	6 (29)	2.2	4 (19)	1.83
Grains and flour ^e^	23	14 (61)	2 (9)	IIa	451	11	2.31	11 (48)	3.74	1 (4)	4.00
Silage	50	11 (22)	2 (4)	ND	ND	ND	4.65	7 (14)	5.77	4 (8)	6.06
Feeding Table	4	2 (50)	0 (0)	-	-	-	-	2 (50)	3.54	0 (0)	-
Silage pit	5	0 (0)	0 (0)	-	-	-	-	0 (0)	-	0 (0)	-
Heap	2	0 (0)	0 (0)	-	-	-	-	0 (0)	-	0 (0)	
Haylage bales	19	8 (42)	1 (5)	-	-	-	2.6	4 (50)	6.00	4 (50)	6.06
Trench	9	0 (0)	0 (0)	-	-	-	-	0 (0)	-	0 (0)	-
Tunnel	1	0 (0)	0 (0)	-	-	-	-	0 (0)	-	0 (0)	-
Other	9	1 (11)	0 (0)	-	-	-	-	1 (11)	5.15	0 (0)	-
Straw	1	0 (0)	0 (0)	-	-	-	-	0 (0)	-	0 (0)	-
Total	141	71 (50)	21 (15)				3.46 ^f^	51 (36)	4.04 ^f^	15 (11)	4.59 ^f^

^a^ CC—Clonal complexes; ^b^ ST—Sequence type; ^c^ MR—Total mixed ration; ^d^ By-products—rapeseed meal (2), potatoes (1), brewer’s spent grains (1); ^e^ *L. fleishmanii*—one grain sample positive with 10 cfu/g; ^f^ The significant differences between the mean counts of *L. monocytogenes*, *L. innocua* and *L. seeligeri* in different types of feeds were not identified (*p* > 0.05); ND—not identified.

**Table 3:** Prevalence of *Listeria* spp. in the soil, feed and water samples from cattle farms.

Sample	No. of Samples	*Listeria* spp.		*L. monocytogenes*			*L. innocua*	*L. seeligeri*
		No. of Positive Sample (%)		Serogroup	ST	CC	No. of Positive Samples (%)	
**Soil**								
Near to pond	3	1 (33)	1 (33)	IIa	37	37	0 (0)	1 (33)
Near to farm	7	4 (57)	3 (43)	IIa	7, 37	7, 37	3 (43)	0 (0)
Near to manure ^a^	15	14 (93)	3 (20)	IIa	451	11	10 (67)	3 (20)
Manure	26	19 (73)	5 (19)	IIa	7	7	15 (58)	2 (8)
Pasture	21	14 (67)	4 (19)	IIa,	18, 37, 1085	18, 37, 1085	12 (46)	1 (4)
Near feed storage	22	15 (68)	2 (9)	IIa	8, 403	8, 403	13 (59)	3 (14)
Bedding at farm	25	16 (64)	5 (20)	IIa	18, 20, 21, 37	18, 20, 21, 37	11 (44)	3 (12)
Straw and bedding in storage	14	8 (57)	1 (7)	IIa	7	7	7 (50)	1 (7)
**Feed** ^a^								
Feeding table	59	40 (68)	14 (24)	IIa, IVb	31,518, 20, 37, 194, 451	11, 18, 20, 27		5 (8)
Pasture	3	1 (33)	0 (0)	-	-	-	1 (33)	0 (0)
Storage	79	29 (37)	7 (9)	IIa	18, 37, 451	11, 18, 371	18 (23)	6 (8)
**Water** ^b^								
Farm								
-drinking bowl	60	45 (75)	13 (22)	IIa	8, 37, 451, 1482	8, 11, 37, 1482	36 (60)	5 (8)
-drinking trough	32	22 (69)	9 (28)	IIa	8, 37, 451, 1482	8, 14, 37, 1482	13 (41)	3 (9)
-other	15	0 (0)	0 (0)	0	-	-	0 (0)	0 (0)
Pasture								
-drinking trough	10	3 (33)	1 (10)	ND	-	-	2 (20)	0 (0)
-water body	7	7 (100)	3 (43)	IIa	18, 37	18,37	1 (14)	2 (28)
Other	8	1 (12)	0 (0)	-	-	-	1 (12)	0 (0)
**Feces**	111	65 (59)	28 (25)	IIa, IIc, IVb	4, 8, 9, 18, 29, 37, 451	4, 8, 9, 11, 18, 29, 37	37 (33)	17 (15)

ST—sequence type; CC—clonal complexes; ^a^ The prevalence of *L. monocytogenes* in soil samples near to manure storage (93%) was significantly higher (*p* < 0.001); ^b^ Differences were not significant differences between the inside and outside environment of the farms for water and soil samples (*p* > 0.05) excluding feed in feed troughs (*p* < 0.05); ND—not identified.

**Table 4:** Genetic diversity of *Listeria monocytogenes* isolates in the environmental and animal samples.

Serogroup	ST/CC	Faeces	Feed	Soil	Water	Total
		No. of Isolates				
IIa	ST7/CC7	0	0	4	0	4
	ST8/CC8	2	0	2	2	6
	ST451/CC11	4	4	2	3	13
	ST399/CC14	0	0	0	1	1
	ST18/CC18	2	4	3	1	11
	ST20/CC20	0	1	1	0	2
	ST21/CC21	0	0	1	0	1
	ST29/CC29	1	0	0	1	2
	ST37/CC37	2	4	7	7	20
	ST403/CC403	0	0	1	0	1
	ST1482/CC1482	0	0	0	2	2
	ST1085/CC1085	0	0	1	0	1
IIc	ST9/CC9	1	0	0	0	0
IVb	ST4/CC4	1	0	0	0	0
	ST194/CC315	0	1	0	0	0

ST—sequence type; CC—clonal complex.

**Table 5:** *Listeria monocytogenes* clonal complexes isolated from case and control cattle farms.

Clonal Complex	Case Farm	Control Farm	Total
	No. of Isolates		
CC4	1	0	1
CC7	2	2	4
CC8	6	0	6
CC9	1	0	1
CC11	5	8	13
CC14	1	0	1
CC18	8	3	11
CC20	0	2	2
CC21	1	0	1
CC29	0	2	2
CC37	7	13	20
CC315	1	0	1
CC403	0	1	1
CC1085	0	1	1
CC1482	0	1	1

CC—clonal complexes.

## Data Availability

All raw sequence reads generated were submitted to the European Nucleotide Archive (http://www.ebi.ac.uk/ena/) under the study accession number PRJEB45227.

## Supplementary Materials

The following are available online at https://www.mdpi.com/article/10.3390/pathogens10070851/s1, Table S1: Questionnaire. Information about the farm.
