# Supplementary material for: Prevalence, Genetic Diversity and Factors Associated with Distribution of Listeria monocytogenes and Other Listeria spp. in Cattle Farms in Latvia
Source: Pathogens. 2021 Jul 6;10(7):851. doi: 10.3390/pathogens10070851 (PMC8308843; doi:10.3390/pathogens10070851)
Supplement: Supplementary file 1 [file pathogens-10-00851-s001.zip › pathogens-1252267-SI.pdf]

Questionnaire

Latvian Council of Science project "Whole genome-based characterization of environmental *Listeria* spp. and their role in ruminants listeriosis and public health", No. lzp-2018/2-0361

The aim: to determine the association between *Listeria spp.* (especially *Listeria monocytogenes*) caused diseases in ruminant herds and animal feed and impact of environmental factors. To find out the possible antimicrobial resistance (AMR) of *Listeria spp.* present at the ruminant herds. To determine the correlation between AMR and used drugs in animal feed and for treatment, and to inform the professionals of the industry about the options and possibilities to reduce the AMR.

Information about the farm

Region and parish

Name of the farm and farm ID number

Type of husbandry: □ industrial (conventional); □ biological farming; □ for own consumption

Animal species at the farm: □ cattle; □ sheep; □ goats

Type of the production: □ milk; □ meat; □ meat and milk

Number of productive animals: □ 20-50; □ 51-200; □ ≥201

Animals at the farm are: □ from the own holding / heard ______%; □ purchased animals _______%

Type of animal housing: □ access to pasture; □ without access to pasture; □ walking area available; □ tie-stall; □ free-stall

Type of soil in pasture:

Housing of young animals: □ special pens inside the shed; □ individual cage inside the shed; □ individual cage outside the shed (started at the age of)

How the manure is removed from shed: □ manually; □ mechanically:
how often (times per) week /month / year

Storage of manure: □ deep barn; □ manure pile; □ in special manure garner; □ outside the shed

Type of bedding: □ peat; □ saw dusts; □ straw; □ sand; □ bedding is not used; □ other

Watering system in the shed: □ automatic drinking trough; □ drinking bowl

Watering system in pasture: □ natural waterbodies (what kind) □ drinking bowl

Feeding and feed type for adult animals: □ silage (what kind); □ hay; □ green pasture; □ grain processing products; □ special mixed feed, including total mixed ratio; □ feed additives; □ other

Type of silage storage: □ in trench; □ in tunnel; □ in bales; □ in a pit; □ other

Do rodents and wild birds can access places where animal feed is stored:

Feeding and feed type for young animals: □ mother's milk till the age of ;
□ milk replacer started at the age __ till _;
□ hay or grass started at the age of_;
□ other___

Origin of the feed: □ industrially produced; □ produced at the farm*; □ other
please, attach the information about feed production process

Feed or water additives: □ are not added; □ organic acid; □ probiotics; □ unknown; □ other

Use of antimicrobials: □ are not used; □ inject individually in case of animal diseases; □ use is not known

Which antimicrobials and in what cases are used for adult cows:
Which antimicrobials and in what cases are used for calves:

Where the antimicrobials are purchased: □ from wholesaler; □ in veterinary pharmacy; □ in pharmacy; □ other

Do animals can access to: □ not productive animals, pets; □ other productive animals of different species (pigs, sheep, goats); □ wild animals and birds; □ small rodents (mice, rats)

Please, identify potential stress factors for the animals at the farm:

Veterinarian at the farm: □ the farm has a contract for permanent veterinary services;
□ the farm has a contract for occasional veterinary services;
□ the farm does not have permanently agreed veterinarian

Use of the disinfectants: □ are not used; □ are used *(please, fill the table)*

Application	Disinfectant, concentration of working solution	Indications	Frequency of the use	Responsible person (position)
Milk line / milking system				
Udder				
Animal feeding and care equipment				
Staff clothing, footwear, hands				
Foot baths				
For the cages of calves				
Feeding utensils/bowels of calves				
Other				

Information about the animal infected with *Listeria spp.*

Species: ☐ cattle; ☐ sheep; ☐ goat

Animal ID number:

Age of the animal: **which lactation:**

Clinical manifestations of listeriosis: ☐ abortion, which month of gestation:

☐ neurological symptoms, specify them

☐ mastitis, shortly describe (how many teats affected)

☐ other

Does the animal previously had abortion or other listeriosis caused diseases: ☐ no

☐ yes (when, what kind)

Does the animal previously had any other diseases: ☐ no

☐ yes (when, what kind of diseases)

Thank you for the response and trust!
